# Development and Characterization of Nanoliposomal Hydroxyurea Against BT-474 Breast Cancer Cells

**DOI:** 10.15171/apb.2020.005

**Published:** 2019-12-11

**Authors:** Azam Akbari, Azim Akbarzadeh, Morteza Rafiee Tehrani, Reza Ahangari Cohan, Mohsen Chiani, Mohammad Reza Mehrabi

**Affiliations:** ^1^Department of Pharmaceutics, Faculty of Pharmacy, Tehran University of Medical Sciences, Tehran, Iran.; ^2^Department of Nanobiotechnology, Pasteur Institute of Iran, Tehran, Iran.

**Keywords:** Breast neoplasms, Drug carriers, Hydroxyurea, Liposomes

## Abstract

***Purpose:*** Hydroxyurea (HU) is a well-known chemotherapy drug with several side effects which limit its clinical application. This study was conducted to improve its therapeutic efficiency against breast cancer using liposomes as FDA-approved drug carriers.

***Methods:*** PEGylated nanoliposomes-containing HU (NL-HU) were made via a thin-film hydration method, and assessed in terms of zeta potential, size, morphology, release, stability, cellular uptake, and cytotoxicity. The particle size and zeta potential of NL-HU were specified by zeta-sizer. The drug release from liposomes was assessed by dialysis diffusion method. Cellular uptake was evaluated by flow cytometry. The cytotoxicity was designated by methyl thiazolyl diphenyl-tetrazolium bromide (MTT) test.

***Results:*** The size and zeta value of NL-HU were gotten as 85 nm and -27 mV, respectively. NL-HU were spherical.NL-HU vesicles were detected to be stable for two months. The slow drug release and Weibull kinetic model were obtained. Liposomes considerably enhanced the uptake of HU into BT-474 human breast cancer cells. The cytotoxicity of NL-HU on BT-474 cells was found to be significantly more than that of free HU.

***Conclusion:*** The results confirmed these PEGylated nanoliposomes containing drug are potentially suitable against in vitro model of breast cancer.

## Introduction

Breast cancer is one of the most prevalent diseases, with a high death rate among women.^1,2^ Current methods for breast cancer therapy are chemotherapy, radiotherapy, and surgery, but all three types of treatment cause high toxicity for patients by killing healthy cells beside cancer cells and leading to severe side effects.^3^ Furthermore, only a little quantity of the drug reaches the target tumor, and most of the drug enters healthy tissues or is rapidly removed.^4^ Among the chemotherapy agents, hydroxyurea (HU) is a well-known, low cost, effective, and safe drug that is extensively applied in the treatment of human cancers.^5,6^ Moreover, this drug has several defects, such as rapid clearance from circulation, low bioavailability, and side effects in patients.^7^ Liposomes are widely used as carriers for delivering anticancer drugs, with multiple approved products for use by patients.^8-12^ In recent years, some researchers have focused on the designing nanosize liposomes with a prolonged circulation time in the blood flow that is believed to increase drug delivery to the tumor. Where nanosize liposomes would pass the tumors vasculature and accumulate in large quantities.-^16^ Several efforts have been made in this field. In a study, Alavi et al prepared and evaluated liposomal hydroxyurea on MCF7 cells.^17^ O’Shaughnessy et al also showed the successful use of PEGylated liposomes containing doxorubicin for breast cancer treatment.^18^ In the present investigation, HU-loaded PEGylated nanoliposomes were prepared and, first, assessed on BT-474 breast cancer cell.

## Materials and Methods

### Materials and cells

Dipalmitoyl phosphatidylcholine (DPPC) and methoxy-polyethylene glycol-derivatized di- stearoyl phosphatidylethanolamine (DSPE-mPEG2000) were obtained from Lipoid GmbH (Lud­wigshafen, Germany). Fluorescein isothiocyanate (FITC), HU and cholesterol (Chol) were provided from Sig­ma-Aldrich (St. Louis, MO, USA), Sucrose, Chloroform, and methanol were prepared from Merck (Darmstadt, Germany). Polycarbonate screens were supplied from Northern Lipids (Vancouver, Canada). Streptomycin-penicillin-glutamine (S/P/G), SephadexG-50 were taken from Invitrogen (Carlsbad, CA, USA). All other chemicals were of analytical grade. As well, the BT-474 cell line was bought from the American Type Culture Collection.

### Preparation of liposomes containing drug

Liposomes were manufactured via the thin-film hydration technique. Briefly, DPPC: Chol: DSPE-mPEG2000 at 7:4:0.18 molar proportions solubilized in a chloroform-methanol mix at a ratio (2:1).

After evaporation of the solvents, the film was dried and suspended in phosphate buffer saline (PBS, pH = 7.4) containing HU (molar ratio drug/lipid = 0.2/1), stirred and warmed up to 50℃ in the water bath. The liposomes were mixed using a homogenizer (Ultra Turrax, NJ, USA) for 4 min at 16 000 rpm and extruded through polycarbonate screens 100 nm for ten circles by an Extruder (Avestin Inc., Ottawa, Canada). The NL-HU were segregated from free HU on a Sephadex G50 column. To determine the encapsulation efficiency (EE), one milliliter of purified NL-HU was disrupted by isopropanol (1:1 ratio) to release the drug.

The value of drug encapsulated in liposomes was computed by a spectrophotometer (UV-1601PC, SHIMADZU, Japan) at 214 nm via plot standard curve and the following formula^19,20^:

$$EE\%\frac{\text{amount of encapsulated HU (mg)}}{\text{amount of total HU (mg)}}\times 100$$

### Phospholipid quantification

The Stewart assay,^21^ was executed to measure the lipid content in the nanoliposomal formulations. Lipids were solubilized in chloroform, followed by removing the solvent by a rotary motion. The thin film was hydrated in DW, 1 mL chloroform added and stored overnight in an oven at 90°C. Then was dissolved by 70 mL chloroform and immediately after centrifugation of the dispersals, we calculated the optical density (OD) of the standard and tests at 485 nm and detected the concentration by the standard curve.

### Size, polydispersity index, and zeta potential

The size, zeta index, and polydispersity value of the NL-HU were examined by a zeta-sizer instrument (Nano ZS3600, Malvern Panalytical Ltd, Malvern, UK) at 25°C in triplicate.

### Morphology of NL-HU

Morphology of NL-HU was assessed by a scanning electron microscopy (SEM, Carl Zeiss EVO LS15, NY, USA) and the transmission electron microscopy (TEM, Zeiss EM 900, Jena, Germany). For SEM analysis, a small quantity of liposomal suspension was scattered on a stub and parched at environment temperature. NL-HU were layered with gold by sputter coater (Nano-Structured Coatings Co. DSR1, Tehran, Iran) that is equipped with a rotary pump to attain vacuum less than 50 militorr, the proper vacuum range for noble metals, and sample coating with an additional thin layer (~10 nm) of the gold conductive material to gather high-quality information from SEM. Finally, were examined by SEM at 26 kV. For TEM study, one droplet of well-dispersed formulations was settled on the carbon-layered copper grid, wiped at environment temperature, and photographed using TEM at 150 kV.

### Drug release, kinetic models and mechanism

To specify the percentage of drug release, 1 mL of NL-HU and free HU were put into the dialysis bags (cut off: 14 000), immersed in 50 mL PBS (pH 7.4) and left on a stirrer for 36 hours at 37℃. At specific time intervals, 1 mL of reservoir buffer was taken and superseded by the same content of the fresh buffer. The amounts of HU released in the PBS were determined by spectrophotometer at 214 nm and the standard curve. The below formula computed the percentage of drug released (Er):

$$Er\%=\frac{V_{0}C_{i}\;+\;V_{0}C_{n}}{M_{HU}}\times 100$$

M_HU_ illustrates the content of HU in the liposomes, V_0_ declares the whole volume of the release media and C_i_ and C_n_ manifest the concentration of HU in *i*th and *n*th aliquot removed, respectively.

To designate the release mechanism from kinetic models including first order, zero order, Weibull, Higuchi, and Korsmeyer Peppas, the release data were analyzed using Excel 2017 (Microsoft Corporation, Redmond, WA, USA) Add-In DD Solver program. The coefficient of determination (R^2^), release rate constants (k_x_), diffusion exponent (n), shape factor (b) were calculated. Well-fit kinetic equation with result data was considered as the best model.

### Stability assessment

Stability of NL-HU was evaluated in buffer PBS at specified intervals within 2 months of storage at 4°C. The zeta potential and size of the specimens were considered as the stability performance.

### Cellular uptake of formulations by flow cytometry

Internalization of the NL-HU and free HU into BT-474 cells was investigated by flow cytometry. Free HU and NL-HU were labeled by FITC. Briefly, 130 μL of PBS (pH = 7.4) and 65 μL of FTIC (2 mg in 1 cc DMSO) were poured into the free HU solution and NL-HU suspension and mixed gently. Unbounded FITC was separated by Sephadex G-50 column. Cells (300 × 10^3^/well) were placed into six-well plates and incubated with the labeled free HU and NL-HU in the incubator at 37°C for 4 hours. The untreated cells were as the control. Finally, the cells were rinsed with cold PBS and appraised using the flow cytometry (Becton Dickinson FACSCalibur, Franklin Lakes, NJ, USA). WinMDI software was used to analyze the results.

### Cytotoxicity assessment

The cytotoxicity of NL-HU against BT-474 cells was designated by MTT assay. Briefly, the cells were kept in RPMI medium containing S/P/G and 10% FBS in a 5% CO2 incubator at 37°C. Then, they were sub-cultured in 96-well plate (50 000 cells/well) in 100 µL of media at 37°C. After 24 hours of incubation, the cells were pasted to the wells. The free HU (as positive control) and NL-HU at the drug concentrations of 200, 400 and 600 µM were poured in the wells and incubated for 48 hours. Then the PBS-containing MTT (5 mg/mL) was added to the treatments and incubated for 3 hours; afterward, the formazan crystals were dissolved in 100 µL of DMSO. The OD was monitored using ELISA reader at 570 nm. Cell viability percentage and IC_50_ (50% inhibitory concentration of cell growth in comparison to untreated control cells as negative control),^22,23^ were determined. All tests were done in triplicate.

### Statistical analysis

Data were shown as mean ± standard deviation from three tests. The results were analyzed in SPSS software (version 20, IBM, Armonk, NY) with the student *t* test and one-way ANOVA followed by Tukey’s HSD post hoc test. Statistical significance was set at *P*< 0.05.

## Results and Discussion

### Preparation and characterization of NL-HU

**Figure 1:**
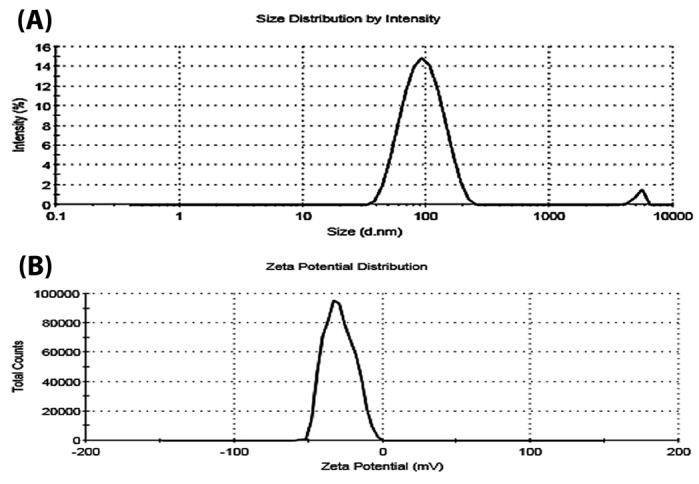
Hydrodynamic size (A) and zeta potentials images (B) of PEGylated liposomes containing Hydroxyurea (NL-HU) by zeta-sizer instrument.

**Figure 2:**
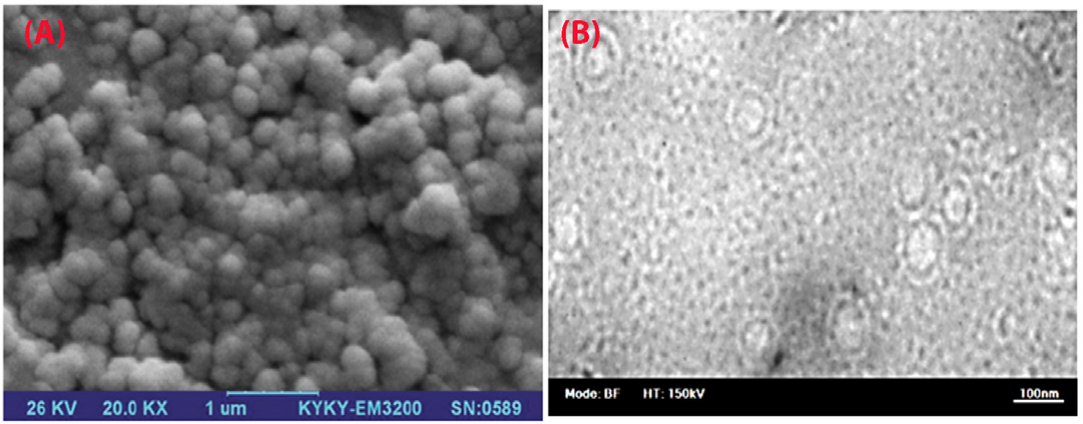
Scanning electron microscopy (A) and Transmission electron microscopy (B) photographs of PEGylated liposomes containing hydroxyurea (NL-HU).

The size of NL-HU was approximately 85 ± 2.2 nm, which was lower than the previous report (Figure 1).^17^ The polydispersity index was equal to 0.12±0.030 for nanoliposomal HU; also, the mean zeta potential of the NL-HU was equal to -27 ± 0.51 mV (Figure 1). NL-HU were spherical, and rather homogenous, as is shown in Figure 2. EE% was equivalent to 88%, which was more than the prior study.^17^ Lipid content of liposomes was equal to 89 ± 3.6%. Particle size and polydispersity index are fundamental factors for the preparation of proper nano-drug delivery systems; they contribute to the toxicity and delivery ability of these systems. For this purpose, the right size should be between 10 and 200 nm, where, the value of 85 nm was in this range due to the suitable choice of constituents, their proportions, and preparation method.^24-28^

Accordingly, the PDI was calculated based on the square of the ratio of standard deviation over diameter; the PDI <0.2 in this study indicates the high homogeneity of the liposomes.^29^ Zeta potential is a significant criterion in the stability of liposome formulations and in blocking their aggregation, as well.^30^ Zeta potential of nanoliposomes was negative because of the existence of carboxylic groups of lipids, drug–to–lipid ratio, pH, and type of lipids used in the liposomes. The obtained zeta potential was enough to keep the stability of liposomal formulations. Based on SEM and TEM images, the NL-HU were found to be spherical due to the appropriate method of liposome preparation. The observed dimensions of liposomes using SEM and TEM were slightly smaller than those of the zeta-sizer because of zeta-sizer displays the hydrodynamic diameter of liposomes, but SEM and TEM depict the dried form of the liposomes.^31^ The EE% of HU was high because of the suitable HU-loading method and drug to lipid ratio.^30,32^

### Profile, kinetic, and mechanism of drug release

**Figure 3:**
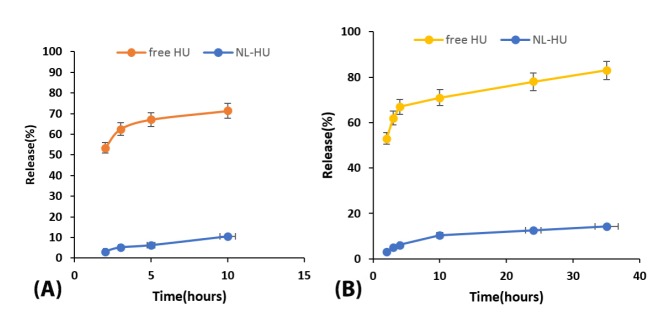
In vitro release of PEGylated liposomes containing Hydroxyurea (NL-HU) and free Hydroxyurea (free HU) in PBS buffer (pH=7.4) at 37°C within first 10 h (A) and 36 h (B).

The pattern of drug release for free HU and NL-HU at pH = 7.4 was presented in Figure 3. Within ten h of drug release at 37°C in the buffer, 67 ± 4.2% of free HU and 11 ± 0.41% of NL-HU were released, respectively. After, the drug release was continued for up to 36 hours that 83 ± 4.8 and 14 ± 1.2% yield release for free HU and NL-HU were obtained, respectively. It is in consistency with the study by Alavi et al.^17^ Drug release from NL-HU was significantly less than free HU because drugs are surrounded by liposomes that prevent them from being released. The liposomes exhibited fast drug release during the first ten hours. The reason for that is HU adsorbed on the liposome or the release of the drug-loaded close to the liposome surface. It was followed by a slow release due to liposome components, the liposome erosion, and HU diffusion mechanisms.^33^ The sustained release pattern from liposomes could diminish prescription times. The mathematical modeling of the release data for NL-HU was done as well, and the kinetic parameters listed in Table 1. Based on our results, the release data of HU from NL-HU within the first 10 hours and up to 36 were found to be in harmony with the Weibull formula. The Weibull equation describes drug dissolution and releases from dosage forms. The factor β in this model is an index of the mechanism of drug transport through the polymer matrix, and Fickian diffusion is the typical release mechanism where β < 0.75. Accordingly, the calculated values of β = 0.72 for first 10 hours and β = 0.51 for up to 36 hours release represented the diffusion mechanism for drug release from nanoliposomes.^34-37^

**Table 1:** Kinetic models parameters of release data

**Sample**	**Model**										
	**Zero order** ***F*** **=** ***k***_0_ ***t***		**First order** ***Ln*** **(1 −** ***F*** **) = −** ***k***_f_ ***t***		**Higuchi** ***F*** **=** ***k***_H_ ***√ t***		**Weibull** ***ln[-ln(1-F*** ***)]=*** ***ln k***_w_ ***+*** ***β*** ***lnt***			**Peppas–Korsmeyer** ***F*** **=** ***k***_kp_ ***t*** ^n^	
	**R** ^2^	**k**	**R** ^2^	**k**	**R** ^2^	**k**	**R** ^2^	**k**	**b**	**R** ^2^	**k**
NL-HU	0.85	0.0031	0.86	0.0034	0.93	0.023	0.94	0.0090	0.51	0.94	0.027
NL-HU (first 10 h)	0.97	0.0086	0.98	0.0093	0.98	0.040	0.98	0.048	0.72	0.97	0.021

Abbreviation: NL-HU: PEGylated nanoliposomes containing Hydroxyurea. Note: R2 is determination coefficient; F is the released drug fractions; k0, kf, kH, kw, and kkp, are constants of the kinetic models; n is the diffusion exponent of the Peppas–Korsmeyer model; β is the shape factor in Weibull model.

### Stability analysis

**Figure 4:**
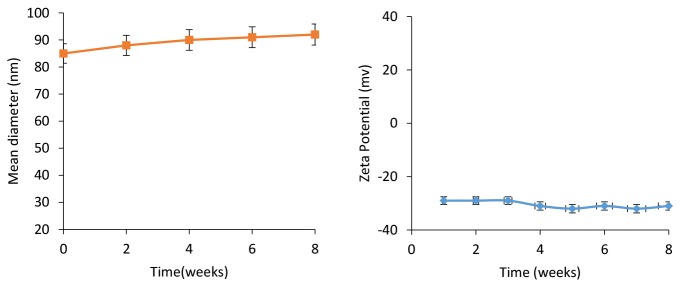
Stability of PEGylated liposomes containing Hydroxyurea (NL-HU) based on mean diameter (A) and zeta potential(B) at 4°C within two months.

As is seen in Figure 4, stability analysis confirmed no considerable changes in the mean zeta potential and particle size for the NL-HU within two months in PBS buffer at 4°C.

These findings were related to the suitability of the applied method, components, and their ratios of liposomes.^38^

### Cellular uptake studies

**Figure 5:**
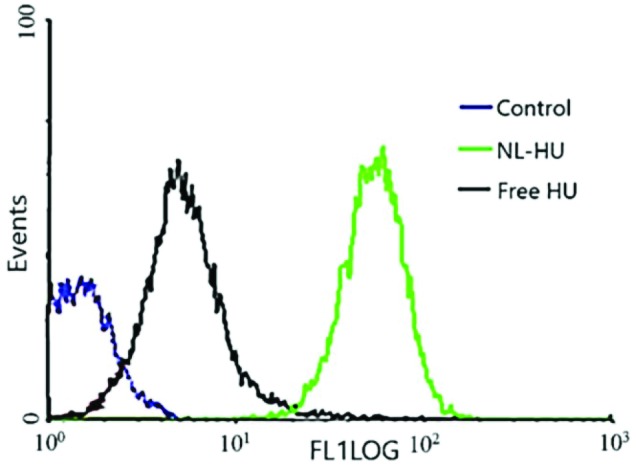
Uptake of PEGylated nanoliposomes containing Hydroxyurea (NL-HU), Hydroxyurea (free HU) and untreated cells control after four h incubation at 37°C with BT-474 cells by flow cytometry.

The results of quantitative cellular internalization analysis by flow cytometry in Figure 5 showed the fluorescence intensity of the NL-HU was remarkably better than that of the free HU in BT-474 cells, which means more uptake of NL-HU compared to free HU in BT-474 cells. It is due to the suitable size of liposomal formulations, type, size of cells, incubation time, and a high tendency between liposome and the cell.^22, 39-42^

### Cell cytotoxicity

**Figure 6:**
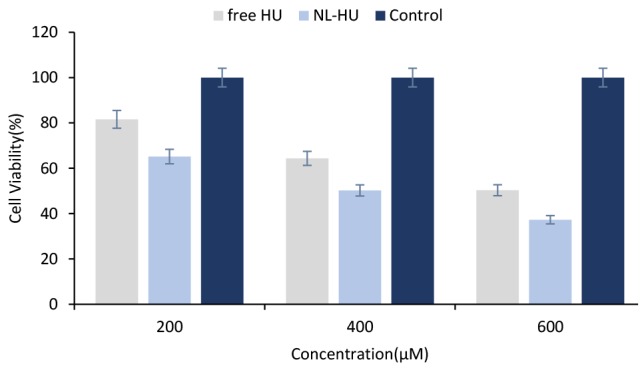
Viability of BT-474 cells with free Hydroxyurea (free HU), PEGylated liposomes containing Hydroxyurea (NL-HU) and negative control at various concentrations after 48 hours.

As is viewed in Figure 6, NL-HU were more toxic to BT-474 cells than free HU, which emphasizes the more anticancer potential of NL-HU against breast cancer than free HU at different concentrations. Cytotoxicity analysis verified less viability and lower IC50 (superior toxicity) of NL-HU (419.29 µM) versus free HU (601.14 µM) at all the concentrations on BT-474 cells; which is attributed to the cell type, exposure time, and liposome charge.^25,43-45^

## Conclusion

We designed and evaluated HU-loaded PEGylated nanoliposomes on an in vitro model of breast cancer. The results demonstrated this liposomal drug system could be potentially useful for delivery of the hydrophilic anticancer drug. Nevertheless, further investigations are needed to assess this formulation on other breast cancer cells, and in vivo.

## Ethical Issues

Not applicable.

## Conflict of Interest

Authors declare no conflict of interest in this study.
